# Age-Associated Capacity to Progress When Playing Cognitive Mobile Games: Ecological Retrospective Observational Study

**DOI:** 10.2196/17121

**Published:** 2020-06-12

**Authors:** Bruno Bonnechère, Jean-Christophe Bier, Olivier Van Hove, Sally Sheldon, Sékou Samadoulougou, Fati Kirakoya-Samadoulougou, Malgorzata Klass

**Affiliations:** 1 Centre de Recherche en Epidémiologie, Biostatistiques et Recherche Clinique Ecole de Santé Publique Université Libre de Bruxelles Brussels Belgium; 2 Department of Psychiatry and Behavioural and Clinical Neurosciences University of Cambridge Cambridge United Kingdom; 3 Department of Neurology Hôpital Érasme Université Libre de Bruxelles Brussels Belgium; 4 Department of Chest and Thoracic Surgery Hôpital Érasme Université Libre de Bruxelles Brussels Belgium; 5 Peak Brain Training London United Kingdom; 6 Evaluation Platform on Obesity Prevention Quebec Heart and Lung Institute Research Centre Quebec City, QC Canada; 7 Laboratory of Applied Biology and Neurophysiology Neuroscience Institute Université Libre de Bruxelles Brussels Belgium

**Keywords:** cognitive performance, brain training, cognitive monitoring, mobile games, aging, serious games

## Abstract

**Background:**

The decline of cognitive function is an important issue related to aging. Over the last few years, numerous mobile apps have been developed to challenge the brain with cognitive exercises; however, little is currently known about how age influences capacity for performance improvement when playing cognitive mobile games.

**Objective:**

The objective of this study was to analyze the score data of cognitive mobile games over a period of 100 gaming sessions to determine age-related learning ability for new cognitive tasks by measuring the level of score improvement achieved by participants of different ages.

**Methods:**

Scores from 9000 individuals of different ages for 7 cognitive mobile games over 100 gaming sessions were analyzed. Scores from the first session were compared between age groups using one-way analysis of variance. Mixed models were subsequently used to investigate the progression of scores over 100 sessions.

**Results:**

Statistically significant differences were found between age groups for the initial scores of 6 of the 7 games (linear trend, *P*<.001). Cognitive mobile game scores increased for all participants (*P*<.001) suggesting that all participants were able to improve their performance. The rate of improvement was, however, strongly influenced by the age of the participant with slower progression for older participants (*P*<.001).

**Conclusions:**

This study provides evidence to support two interesting insights—cognitive mobile game scores appear to be sensitive to the changes in cognitive ability that occur with advancing age; therefore, these games could be a convenient way to monitor cognitive function over long-term follow-up, and users who train with the cognitive mobile games improve regardless of age.

## Introduction

According to the World Health Organization, the proportion of the population aged 60 years and older will double by the year 2050 to an estimated 2 billion people [1]. The two major health-related problems that have been linked to increased life expectancy are an increased risk of falling due to age-related changes in musculoskeletal and neural systems [2] and cognitive decline due to age-related changes in the brain [3]. In the past decade, the use of video games in rehabilitation for various pathological conditions [4] and for improving balance in older adults [5] has become more and more popular.

While healthy aging is associated with some progressive decline in cognitive function, especially in processing speed and executive functions [6], pathological conditions related to aging may abnormally affect cognitive function leading to mild cognitive impairment or dementia. Globally, the number of people with dementia is estimated to be 50 million and with nearly 10 million new cases every year, represents a significant public health problem. The negative impact of cognitive disease on patients, relatives, and nations is a major public health problem which must be addressed [7,8].

Although aging has been shown to be associated with reduced brain plasticity due to structural and functional changes in the brain [9-11]; processing speed, executive function, and memory are the cognitive functions that are most affected by ageing [12]. Numerous cognitive intervention studies have documented that performance improvements during cognitive tasks are maintained until very late in life [13].

The use of video games that are specifically developed to train and challenge the brain (ie, cognitive games) have been popularized by games such as Dr. Kawashima’s How Old Is Your Brain [14] and have attracted the interest of adults of different ages. Gamification of cognitive assessment and cognitive training have gained interest in the medical community [15] to increase patient participation and alleviate boredom. The potential of such cognitive mobile games to decelerate the cognitive decline associated with ageing has led to an increase in their use by patients and older adults [16].

In a systematic review [17] that analyzed the efficacy of computerized cognitive training in healthy older adults, it was concluded that this type of intervention was modestly effective at improving cognitive performance in healthy older adults, but also that efficacy varied across cognitive domains. Similarly, a recent Cochrane review [18] found low-quality evidence that suggested that, in healthy older adults, immediately after completion of 12 or more weeks of cognitive training using computerized solutions, small benefits may be seen for global cognitive function when compared with active controls and for episodic memory when compared with an inactive control. This absence of effect is not specific to cognitive mobile games; evidence supporting the benefits of cognitive training programs on functional abilities are sparse, thus warranting further research to identify effective interventions [19].

Mobile technology has spread rapidly around the globe. Today, it is estimated that more than 5 billion people own a mobile device, over half of which are smartphones [20]. As the use of mobile health apps and wearable sensors increases, the impact of digital health technology on patient care increases likewise. In 2017, the number of health-related mobile apps available to consumers surpassed 318,000—nearly double the number that was available just two years prior [21]. This significant increase in the use and availability of health-related devices and apps offers interesting prospects in the medical field, among others, for monitoring and training cognitive functions. Proof-of-concept studies have shown that smartphone-based cognitive testing seems promising for large-scale data collection in population studies [22].

Despite the gain in popularity of cognitive games in both young and older adults, age-related changes in performance with frequent use of cognitive mobile games in an ecological study environment have not yet been investigated.

The aim of this study was to evaluate if cognitive mobile games are useful for monitoring and training cognitive functions in subjects of different ages by (1) assessing if game scores are adequately able to differentiate between different age groups and (2) comparing the trajectories of scores over 100 game sessions to evaluate the extent that people of different ages can improve their cognitive performance.

## Methods

### Study Design and Participants

This study was a retrospective observational study which used the anonymized data of 9000 individuals. This study was approved by the Cambridge Psychology Research Ethics Committee (Pre.2020.28) and all participants had agreed that their data could be used for research purposes when installing the app. Data from individuals who met the inclusion criteria (having completed 100 sessions of training with 7 specific cognitive mobile games) were randomly selected from a database provided by Peak Brain Training [23]. Descriptions of the cognitive mobile games that are included in the Peak app are presented in Multimedia Appendix 1. To select the data, simple random sampling was used; 65,428 data sets met the criteria, but a subset of participant data were selected (N=9000) to have the same numbers of participants in each age group (n=1500 per group).

Anonymized scores from the cognitive mobile games which had been automatically recorded by the app were analyzed for each of the six groups: ages 18 to 24 years, mean 21.25 (SD 2.16) years; 25 to 34 years, mean 30.57 (SD 3.28) years; 35 to 44 years, mean 40.29 (SD 4.17) years; 45 to 54 years old, mean 49.42 (SD 3.31) years), 55 to 64 years, 59.66 (SD 3.49) years; and 65 years or older, mean 70.50 (SD 4.16) years. The time of the training was 13 hours (ie, time doing exercises which did not include the pauses between the different cognitive mobile games) and the median duration of the training was 204 (104-346) days with no significant differences between the age groups.

### Procedures

**Figure 1:**
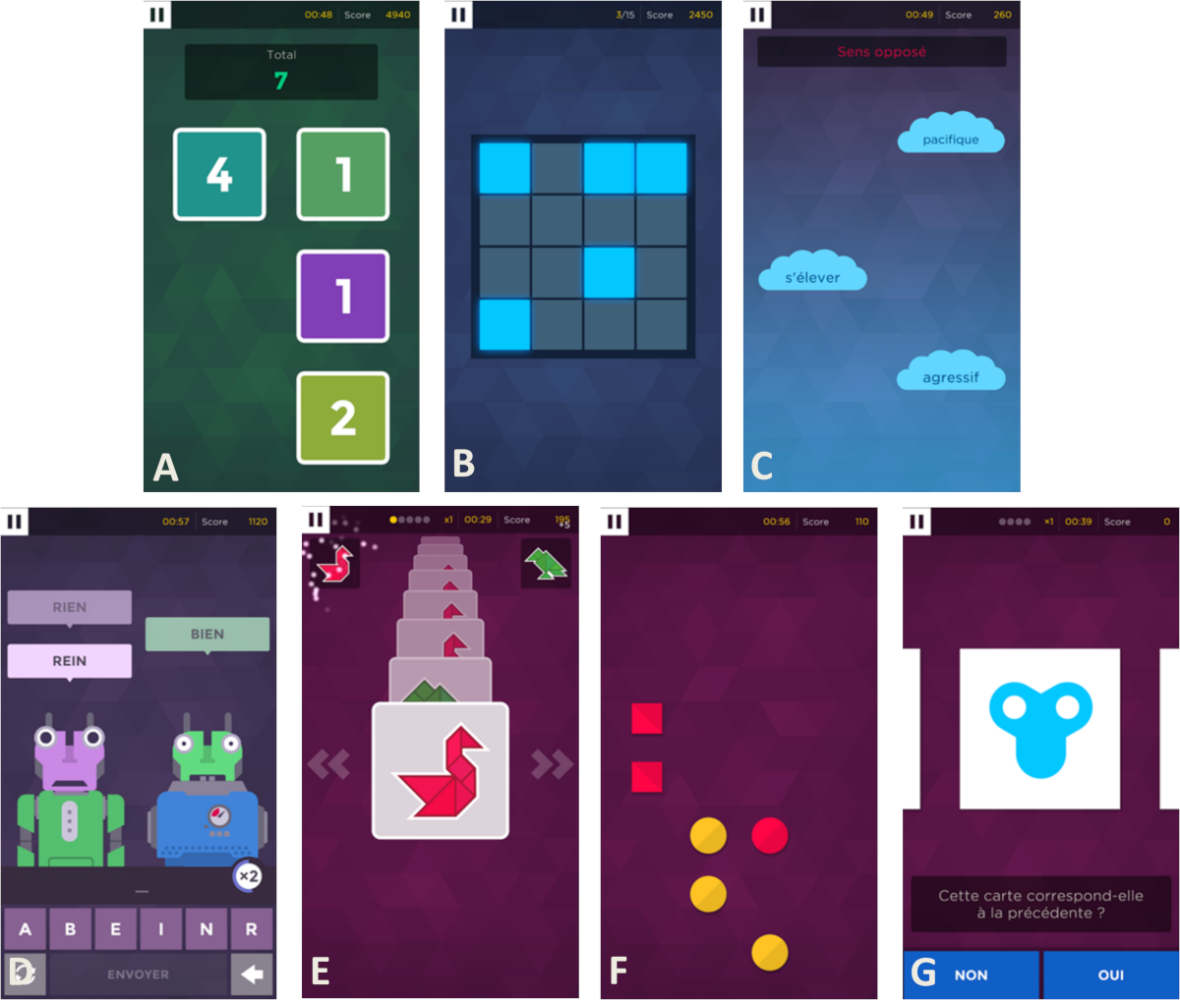
Screenshots of the 7 cognitive mobile games selected for this study from the Peak App. A) Square Numbers, B) Memory Sweep, C) Word Pair, D) Babble Bot, E) Must Sort, F) Unique, G) Rush Back.

Cognitive mobile games (Square Number, Memory Sweep, Word Pair, Babble Bot, Must Sort, Unique, and Rush Back) were used to evaluate the time course of game scores over 100 gaming sessions. The games were organized into categories based upon which main cognitive function they focused. The 7 cognitive mobile games were selected based on a previous study [24] that identified correlations between scores obtained for these 7 particular games and scores in two clinical cognitive assessments (Mini-Mental State Examination and Addenbrooke Cognitive Evaluation) in elderly subjects with and without cognitive impairments [24]. Screenshots of the 7 cognitive mobile games are presented in Figure 1. Full descriptions are presented in Multimedia Appendix 1. The cognitive mobile games could be played on a smartphone or tablet. The scores for 100 sessions were included in the analysis. One session was defined as the completion of one level in the cognitive mobile games. Descriptive session data (mean duration of one session and total training duration) are presented in Multimedia Appendix 2.

### Outcomes

The main outcome was the score obtained for each session for each of the 7 games for each age group. Each cognitive mobile games had its own scoring system which is described in Multimedia Appendix 1.

### Statistical Analysis

For each game, scores from the first session were compared between age groups using one-way analysis of variance (ANOVA) to determine if age had an influence on initial score. Omega-square was used to estimate effect size [25]. Bonferroni tests were used to correct for multiple comparisons in posthoc analysis as well as to test for linear trends.

Then, for each cognitive mobile game, a mixed model with random intercept was used. The scores from each session were treated as repeated measures. The model equation is

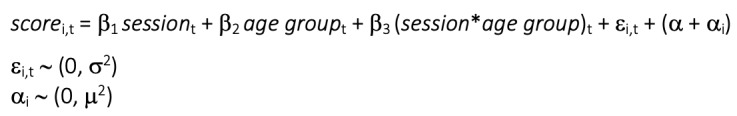



where α and β_1, 2, 3_ represent fixed effect; ε_i,t_ represents random error, and α_i_ represents the measure of the random effect. Fixed effects of age group, session (1 to 100), and the interaction between age group and session were specified. The estimated baseline measures were constrained to be identical in every age groups by subtracting the mean values of each group’s first session from all the sessions. This approach was equivalent to adjusting for baseline and permitting the relationship between baseline and follow-up scores to differ at each session. The fact that the explanatory variables were centered using the mean values allowed this effect to be directly interpreted as an intergroup effect [26].

Likelihood ratio tests were used to test the significance of the random effects model and the linear mixed model with interaction. Since we observed a large range in the duration to complete the 100 sessions, we also performed univariate linear regression to assess if the duration of the intervention had an influence on the outcome.

Statistical analyses were performed at an overall significance level of .05 and were carried out in RStudio (version 1.1.442) using R (version 3.4.4) and in STATA statistical software (release 13; StataCorp LLC).

## Results

### Initial Scores

First session scores differed significantly between age groups for each game (*P*<.001 for each; Square Number, Memory Sweep, Word Pair, Babble Bot, Must Sort, and Rush Back) with the exception of Unique (*F*_5, 8994_=1.2, *P*=.29), a game which addressed visual attention and recognition. The results of the ANOVA are presented in Table 1. Differences between age groups and posthoc tests are presented in Multimedia Appendix 2. Interestingly, we observed a linear decrease in initial scores with increasing age for all cognitive mobile games except Word Pairs (*P*=.29). In Word Pairs (semantic access), the group aged 65 and older outperformed all other age groups; a linear trend in the opposite direction was identified (*P*<.001) indicating a lower score for younger participants.

**Table 1:** One-way ANOVA and effect size results for each cognitive mobile game.

Cognitive mobile games	*F* test (*df1*,*df2*)	*P* value	Effect size^a^
Square Number	15.4 (5, 8994)	<.001	0.08
Memory Sweep	534.6 (5, 8994)	<.001	0.23
Word Pair	14.1 (5, 8994)	<.001	0.01
Babble Bot	30.0 (5, 8994)	<.001	0.02
Must Sort	15.9 (5, 8994)	<.001	0.01
Unique	1.2 (5, 8994)	.29	N/A^b^
Rush Back	239.8 (5, 8994)	<.001	0.12

^a^small effect size was <0.01, medium effect size was >0.01 and <0.06, and large effect size was >0.14

^b^N/A: not applicable.

### Changes in the Scores

**Figure 2:**
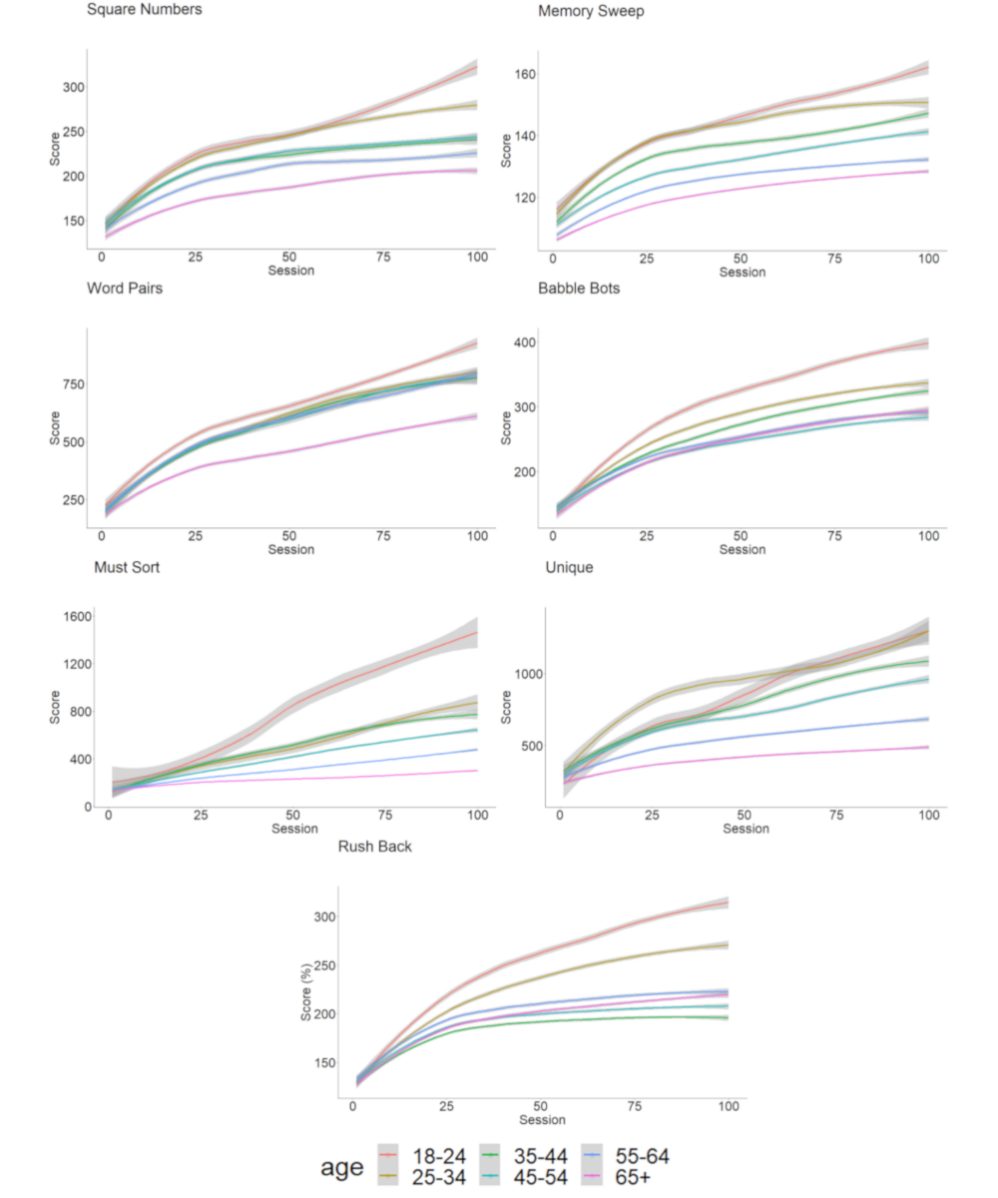
Time courses of the normalized score through the 100 sessions for the different age groups. Grey bands indicate 95% confidence intervals.

**Figure 3:**
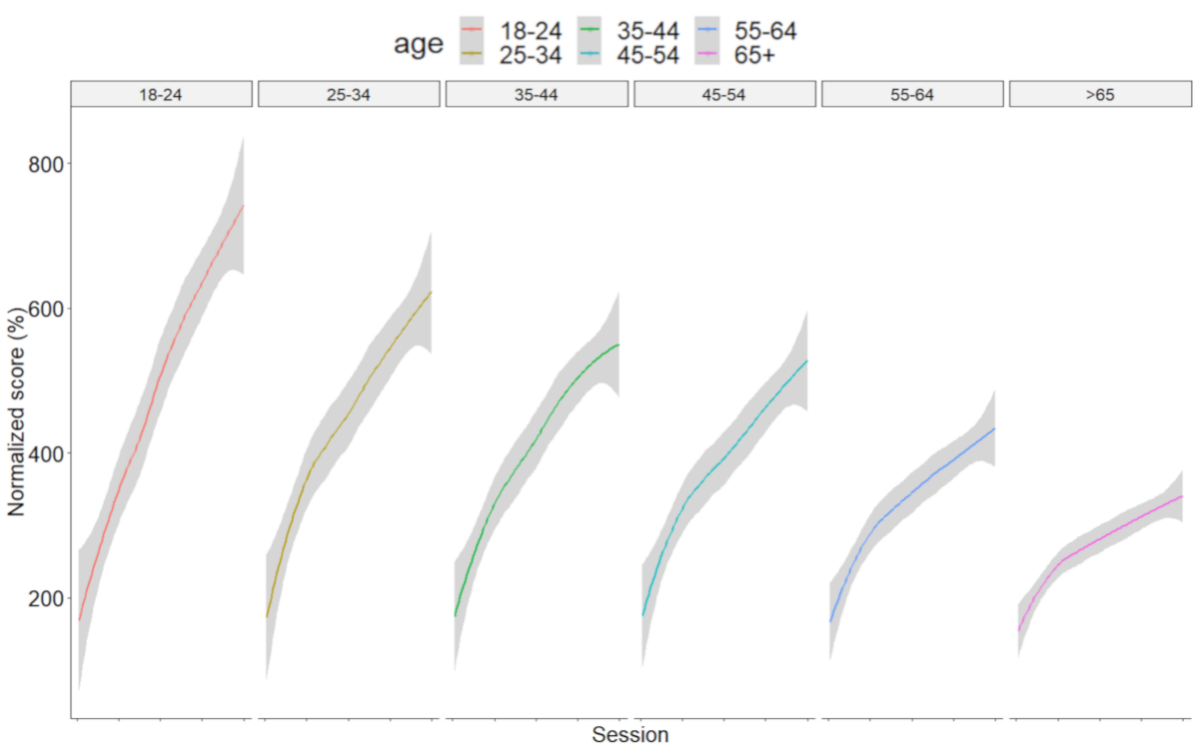
Mean normalized results of the 7 cognitive mobile games for the different age groups. *Normalized results*_i_= 100 + [(*Results*_i_-*Results*_1_/*Results*_1_) × 100]. Grey bands indicate 95% confidence intervals.

The time course of scores over the 100 sessions are presented in Figure 2 and the results of the mixed model for the interaction between the session and age group are presented in Table 2. Complete results of the mixed model are presented in Multimedia Appendix 3. An important findings of this analysis was the interaction between training session and the age (*P*<.001). A linear trend was found for the interaction between age and session indicating that all age groups improved regardless of which cognitive mobile game was played, but that improvement progress was slower for older participants. To better visualize the influence of age on learning capacity over the course of playing the games, we plotted the normalized results of the 7 cognitive mobile games on separate plots in Figure 3.

**Figure 4:**
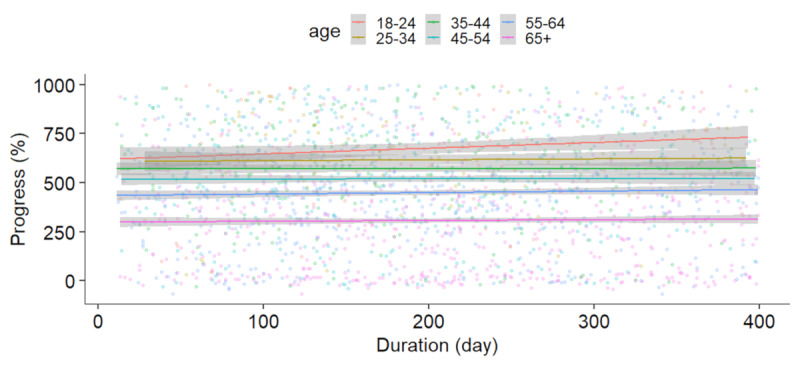
Linear regression between the duration of the period to perform the 100 sessions and the mean progress in the 7 cognitive mobile games for the different age groups (β = 0.02, SE = 0.06, *P*=.71).

No significant correlation was found between the duration of the period for 100 sessions and progress in the games for any of the age groups. Results of the linear regression for the mean progress of the 7 cognitive mobile games and duration of the total training period are presented in Figure 4.

**Table 2:** Results of the mixed effect model analysis for the interaction between session and age group for each cognitive mobile game.

Cognitive mobile game	β_3_ (95% CI)				
	25-34 years	35-44 years	45-54 years	55-64 years	≥ 65 years
Square Numbers	–84 (–93, –76)	–147 (–155, –139)	–191 (–199, –183)	–231 (–239, –223)	–274 (–281, –266)
Memory Sweep	–99 (–104, –94)	–158 (–163, –153)	–207 (–212, –202)	–241 (–245, –237)	–250 (–254, –245)
Word Pair	–6 (–12, 1)	–44 (–49, –39)	–84 (–89, –79)	–111 (–116, 107)	–147 (–152, 143)
Babble Bots	–47 (–50, –43)	–77 (–81, –73)	–102 (–106, –99)	–117 (–121, –113)	–124 (–128, –120)
Must Sort	–68 (–77, –59)	–305 (–314, –297)	–524 (–532, –516)	–683 (–690, –675)	–812 (–819, –804)
Unique	–79 (–84, –74)	–191 (–195, –186)	–270 (–275, –265)	–327 (–332, –322)	–392 (–397, –388)
Rush Back	–82 (–88, –76)	–280 (–285, –275)	–407 (–412, –401)	–478 (–482, –472)	–510 (–515, –505)

## Discussion

### Principal Findings

This study, using a large sample in an ecological environment, found that lower scores are obtained during the first session in older age groups and that slower rates of progression were apparent in the cognitive mobile game scores of older age groups.

The lower initial score for 6 out of the 7 cognitive mobile games in the older groups is in agreement with the literature that suggests a decline of most cognitive functions—processing speed, memory, visuospatial skills, and executive functions—with advancing age [27]. In interpreting our results, the following considerations must, however, be taken into account. Age-related decline can affect other functions. It has been demonstrated that other processes such as slower reaction speed [28],‎ poorer vision [29], and slower motion ‎ [30] with advancing age and could also affect performance in cognitive mobile games. In addition, familiarity with touchscreen tools is generally lower in the elderly population [31]. Therefore, the observed differences in initial cognitive mobile game scores between groups may not be due solely to the changes in cognitive function that occur with advancing age. This observation was supported by the nonsignificant effect of age on the first session score for the cognitive mobile game Unique. This game required visual attention and recognition skills, but was less dependent on reaction time, dexterity, and familiarity with mobile devices compared to the other cognitive mobile games.

Our findings, both for the initial scores and the evolution over time, are in agreement with those of a previous study [27] indicating that not all cognitive functions are affected by age in the same way; simple attention, semantic knowledge, vocabulary, and autobiographical remote memory appear to be more resistant to the regressive effects of aging [27]. A longitudinal study [32] showed that a decline in the ability to rapidly process information and to invoke executive processes occurs across the lifespan and is more pronounced after the age of 60. This is similar to what was observed for Must Sort and Rush Back. In contrast, semantic memory and short-term memory have shown remarkable preservation across most of the adult lifespan, with declines occurring only very late, and not systematically, in life [9]. On the other hand, some functions seem to improve with normal aging, such as semantic memory and richness of vocabulary [33] as well as verbal abilities [27], which is in line with the better scores that were observed for Word Pair in older age groups compared to those of the younger age groups but that were not observed in Babble Bot. Although both cognitive mobile games focus on verbal abilities, the nature of the task in each is quite different: Word Pair challenges semantic memory and understanding of the words to pair them, while Babble Bots required a good knowledge of word spelling to form words based on random letters. This observation is therefore in agreement with the literature showing that retrieval of the meaning of words and other semantic processes are preserved whereas written and spoken spelling abilities are affected by ageing [34].

While changes in the different cognitive abilities over the lifespan have been relatively well documented [3,35,36], there has been less evidence on the ability to learn new cognitive tasks across the lifetime [37,38]. Our findings showed that cognitive mobile game scores increased in all age groups demonstrating that, although the older population is generally considered to be less familiar with the use of touchscreen technology [30], adults aged 65 and older are nevertheless able to benefit from this mobile game training to improve their cognitive performance. Despite slower progress being evident in older age groups, the results demonstrated that cognitive function remains plastic across the lifespan [39]

### Strengths and Limitations

The strengths of this study were the large sample size and that results were obtained in an ecological environment, which increases external validity.

The main limitation of this study was that only scores from the cognitive mobile games were used to assess the progression, and information about the cognitive health or the level of education of the participants were not collected, nor was the level of attention and concentration of the participants during the exercises known. Furthermore, due to the design of the study which used longitudinal analysis of the scores from cognitive mobile games performed by real users of the app, we did not have control groups. Since the results of the cognitive mobile games were used as the outcome to assess the participants, we indirectly evaluated a set of the two to three cognitive abilities used for each cognitive mobile game, rather than one specific cognitive function [40].

Also, due to the retrospective design of this study, we must acknowledge that most of the participants of this study were probably quite familiar with new technologies [41] and that our results may be not generalizable to the general older population.

### Future Works

Little is currently known about whether cognitive skill improvements from playing cognitive mobile games are transferable to real-life, which represents a major challenge in this field of research [39]. Further studies should, therefore, try to elucidate the possibility of transfer between cognitive mobile games performance and activities of daily living.

One study [42], conducted over a 10-year period, suggested that the risk of dementia could be reduced in individuals who took part in a computerized cognitive training that aimed to improve speed of processing. This observation is encouraging, but more long-term longitudinal studies are needed to determine if cognitive mobile games can be used to slow or detect early signs of cognitive decline [43,44].

Further studies are also needed to evaluate how the scores obtained in the cognitive mobile games correlate with clinical neuropsychological assessments and cognitive functioning in everyday life in patients with various pathologies or dementia [45].

In general, the use of cognitive mobile games as digital biomarkers for real-life monitoring of cognitive functions in apparently healthy subjects and patients requires further investigation to evaluate its feasibility (as well as in subjects unfamiliar with smartphones technology) and to determine which outcomes are best correlated with cognitive decline [46].

### Conclusions

Our results show that the initial scores of most of the cognitive mobile games were lower for older age groups, as expected from a physiological viewpoint. The rate of the cognitive performance progress, as measured by changes in the cognitive mobile game scores, is dependent on age, but participants of all ages were able to improve their performance.

These findings suggest that cognitive mobile game scores are sensitive to changes in the cognitive abilities that occur with advancing age and that all age groups can learn new skills using mobile technology. These encouraging results open up the possibility of using cognitive mobile games to simultaneously train and monitor cognitive functions, facilitating cheaper and more regular follow-up. With clinicians facing increasing financial and time constraints, and therefore, less time for face-to-face consultations with patients [43], this type of monitoring might be of particular benefit to detect early signs of cognitive decline and to offer more efficient preventive interventions. Additional research is required to test the validity and feasibility of this kind of approach before it can be applied to clinical practice.

## Appendix

Multimedia Appendix 1 Instructions, cognitive abilities trained and scoring system of the CMG included in this study.

Multimedia Appendix 2 Time per session and total training time for each CMG.

Multimedia Appendix 3 Results of the one-way ANOVA and post-hoc analyses, ω2 is the measure of the effect size (ω2 < 0.01 = small, between 0.01 and 0.06 = medium, ω2 > 0.14 = large) – and scores differences [95% confidence interval] between age group (the youngest groups are taken as reference values).

Multimedia Appendix 4 Results of the mixed effect models analysis. ß coefficient [95% CI].

## Abbreviations

ANOVA: analysis of variance
