# Supplementary material for: Age-Associated Capacity to Progress When Playing Cognitive Mobile Games: Ecological Retrospective Observational Study
Source: JMIR Serious Games. 2020 Jun 12;8(2):e17121. doi: 10.2196/17121 (PMC7320308; doi:10.2196/17121)
Supplement: Multimedia Appendix 1 [file games_v8i2e17121_app1.docx]

## Multimedia Appendix 1: Instructions, cognitive abilities trained and scoring system of the CMG included in this study.

| **CMG** | **Instruction** | **Cognitive abilities trained** | **Scoring system** |
| --- | --- | --- | --- |
| **Square Numbers**  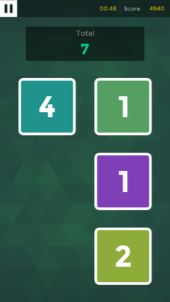 | Match the target shown on top by adding two or more number blocks | - Quantitative reasoning  - Arithmetic  - Working memory | Base score for each correct answer with a speed related bonus (50 - (elapsed seconds ^$^ 5)).  Streak up after 4 correct rounds, down after 5 incorrect. |
| **Memory Sweep**  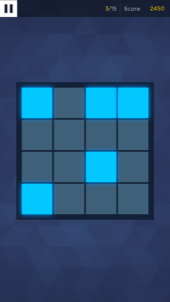 | Memorize the positions of the highlighted tiles and remind their positions when gone | - Attention  - Spatial memory  - Working memory | Points for each correct square (250) plus a base score for complete round.  Streak up after 1 correct answer, down after 1 incorrect moves. |

**Multimedia Appendix 1 (continued)**

| **Word Pair**  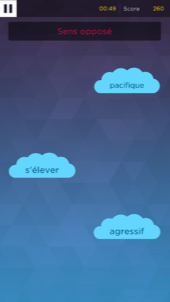 | Pair words according to a specific rule presented (similar, opposite) | - Semantic access  - Vocabulary | Base score for correct round, Streak up after 2 correct answers, down after 2 incorrect. |
| --- | --- | --- | --- |
| **Babble Bots**  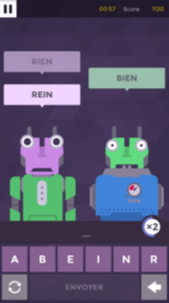 | Create the maximal number of words of at least 3 letters with the 6 proposed letters | - Word fluency  - Vocabulary | Points for letters in word multiplied by the word length, the streak multiplier. Letter scores are localised to the region based on the Scrabble scoring system |

**Multimedia Appendix 1 (continued)**

| **Must Sort**  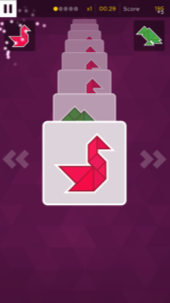 | Sort the items correctly by tapping on the right side | - Response control  - Task shifting | Base score multiplied by streak multiplier, streak is incremented by correct answers and is reduced to 1 on incorrect or more than 5 seconds between answers. |
| --- | --- | --- | --- |
| **Unique**  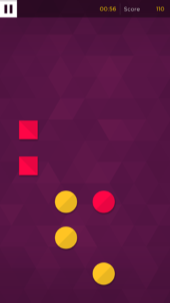 | Find the odd one out and tap on it | - Visual attention  - Visual recognition | Baseline score per correct answer based on difficulty level. Delta is added to the baseline and becomes larger with consecutive correct answers.  (Baseline + (streak ^$^ delta)) Streak of 8 correct up, 6 down. |

**Multimedia Appendix 1 (continued)**

| **Rush Back**  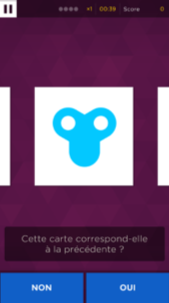 | Memorize a shape, then decide if the next shape matches the one memorized. | - Sustained attention  - Visual recognition  - Working memory | One base score per difficulty with a multiplier which goes up and down based on streak.  Streak up of 4 correct in a row but not changed during game play  Bonus for end of game, current streak multiplier ^$^ bonus |
| --- | --- | --- | --- |
