# Supplementary material for: Age-Associated Capacity to Progress When Playing Cognitive Mobile Games: Ecological Retrospective Observational Study
Source: JMIR Serious Games. 2020 Jun 12;8(2):e17121. doi: 10.2196/17121 (PMC7320308; doi:10.2196/17121)
Supplement: Multimedia Appendix 2 [file games_v8i2e17121_app2.docx]

**Multimedia Appendix 2: Time per session and total training time for each CMG.**

| **CMG** | **Time per session (s)** | **Total time (min)** |
| --- | --- | --- |
| Square Numbers | 70 | 117 |
| Memory Sweep | 90 | 150 |
| Word Pair | 90 | 150 |
| Babble Bots | 60 | 100 |
| Must Sort | 45 | 75 |
| Unique | 70 | 117 |
| Rush Back | 45 | 75 |
