# Supplementary material for: Age-Associated Capacity to Progress When Playing Cognitive Mobile Games: Ecological Retrospective Observational Study
Source: JMIR Serious Games. 2020 Jun 12;8(2):e17121. doi: 10.2196/17121 (PMC7320308; doi:10.2196/17121)
Supplement: Multimedia Appendix 3 [file games_v8i2e17121_app3.docx]

## Multimedia Appendix 3: Results of the one-way ANOVA and post-hoc analyses, ω^2^ is the measure of the effect size (ω^2^ < 0.01 = small, between 0.01 and 0.06 = medium, ω^2^ > 0.14 = large) – and scores differences [95% confidence interval] between age group (the youngest groups are taken as reference values).

| **Square Numbers, ANOVA (F(5,8994) = 15·36, p < 0·001, ω^2^ = 0·08)** | | | | | |
| --- | --- | --- | --- | --- | --- |
| Age group | 18 – 24 | 25 – 34 | 35 – 44 | 45 – 54 | 55 – 64 |
| 25 – 34 | 234  [-884 ; 1352] |  |  |  |  |
| 35 – 44 | 303  [-815 ; 1421] | 69  [-1050 ; 1188] |  |  |  |
| 45 – 54 | 231  [-886 ; 1348] | -3  [-1121 ; 1115] | -72  [-1190 ; 1045] |  |  |
| 55 – 64 | **-1354**  **[-2467 ; -241]^$$^** | **-1588**  **[-2702 ; -475]^$$$^** | **-1657**  **[-2771 ; -544]^$$$^** | **-1585**  **[-2697 ; -473]^$$$^** |  |
| ≥ 65 | **-2285**  **[-3402 ; -1168]^$$$^** | **-2519**  **[-3637 ; -1401]^$$$^** | **-2588**  **[-3706 ; -1471]^$$$^** | **-2516**  **[-3633 ; -1399]^$$$^** | -930  [-2042 ; 180] |
| **Memory Sweep, ANOVA (F(5, 8994) = 534·6, p < 0·001, ω^2^ = 0·23)** | | | | | |
| Age group | 18 – 24 | 25 – 34 | 35 – 44 | 45 – 54 | 55 – 64 |
| 25 – 34 | **-1722**  **[-2270; -1174] ^$$$^** |  |  |  |  |
| 35 – 44 | **-3647**  **[-4195 ; -3099] ^$$$^** | **-1924**  **[-2472; -1377] ^$$$^** |  |  |  |
| 45 – 54 | **-5338**  **[-5886; -4790] ^$$$^** | **-3615**  **[-4163; -3068] ^$$$^** | **-1691**  **[-2238; -1143] ^$$$^** |  |  |
| 55 – 64 | **-7022**  **[-7568; -6477] ^$$$^** | **-5300**  **[-5846; -4754] ^$$$^** | **-3375**  **[-3921; -2829] ^$$$^** | **-1684**  **[-2229; -1138] ^$$$^** |  |
| ≥ 65 | **-8187**  **[-8734; -7640] ^$$$^** | **-6464**  **[-7011 ; -5917] ^$$$^** | **-4539**  **[-5086; --3992] ^$$$^** | **-2848**  **[-3395; -2301] ^$$$^** | **-1164**  **[-1709; -619] ^$$$^** |

**Multimedia Appendix *3 (continued)***

| **Word Pair, ANOVA (F(5, 8994) = 14·06, p < 0·001, ω^2^ = 0·01)** | | | | | |
| --- | --- | --- | --- | --- | --- |
| Age group | 18 – 24 | 25 – 34 | 35 – 44 | 45 – 54 | 55 – 64 |
| 25 – 34 | -175  [-662; 310] |  |  |  |  |
| 35 – 44 | 50  [-436; 536] | 226  [-260; 712] |  |  |  |
| 45 – 54 | 336  [-149; 822] | **512**  **[26; 998]^$^** | 286  [-199; 772] |  |  |
| 55 – 64 | 366  [-118; 851] | **542**  **[57; 1027]^$^** | 316  [-168; 801] | 30  [-454; 514] |  |
| ≥ 65 | **1098**  **[612; 1584] ^$$$^** | **1274**  **[788; 1759] ^$$$^** | **1048**  **[562; 1533] ^$$$^** | **761**  **[276; 1246] ^$$$^** | **731**  **[247; 1216] ^$$$^** |
| **Babble Bots, ANOVA (F(5, 8994) = 30·01, p < 0·001, ω^2^ = 0·02)** | | | | | |
| Age group | 18 – 24 | 25 – 34 | 35 – 44 | 45 – 54 | 55 – 64 |
| 25 – 34 | **1512**  **[444; 2581] ^$$$^** |  |  |  |  |
| 35 – 44 | **1331**  **[263 ; 2399] ^$$$^** | -181  [-1249; 887] |  |  |  |
| 45 – 54 | 592  [-474; 1659] | -919  [-1987; 147] | -738  [-1806; 328] |  |  |
| 55 – 64 | -762  [-1825; 300] | **-2774**  **[-3338; -1211] ^$$$^** | **-2093**  **[-3156; -1031] ^$$$^** | **-1354**  **[-2416; -293] ^$$$^** |  |
| ≥ 65 | **-2339**  **[-3404; -1275] ^$$$^** | **-3852**  **[-4917; -2787] ^$$$^** | **-3671**  **[-4735; -2060] ^$$$^** | **-2932**  **[-3996; -1868] ^$$$^** | **-1577**  **[-2636; -518] ^$$$^** |

**Multimedia Appendix *3 (continued)***

| **Must Sort, ANOVA (F(5, 8994) = 15·91, p < 0·001, ω^2^ = 0·01)** | | | | | |
| --- | --- | --- | --- | --- | --- |
| Age group | 18 – 24 | 25 – 34 | 35 – 44 | 45 – 54 | 55 – 64 |
| 25 – 34 | -445  [-936; 46] |  |  |  |  |
| 35 – 44 | **-556**  **[-1047 ; -64]^$^** | -110  [-602; 380] |  |  |  |
| 45 – 54 | **-796**  **[-1276; -295] ^$$$^** | -340  [-831; 150] | -230  [-720; 260] |  |  |
| 55 – 64 | **-1092**  **[-1581; -604] ^$$$^** | **-647**  **[-1136; -158] ^$$^** | **-536**  **[-1025; -48]^$^** | -306  [-794; 181] |  |
| ≥ 65 | **-1358**  **[-1847; -869] ^$$$^** | **-913**  **[-1402; -424] ^$$$^** | **-802**  **[-1291; -313] ^$$$^** | **-572**  **[-1061; -84] ^$$$^** | -266  [-752; 220] |
| **Unique, ANOVA (F(5, 8994) = 1·241, p =0·287)** | | | | | |
| Age group | 18 – 24 | 25 – 34 | 35 – 44 | 45 – 54 | 55 – 64 |
| 25 – 34 | -346  [-1016 ; 322] |  |  |  |  |
| 35 – 44 | -246  [-915 ; 423] | 100  [-569 ; 769] |  |  |  |
| 45 – 54 | -292  [-961 ; 376] | 54  [-614 ; 723] | -46  [-715 ;623] |  |  |
| 55 – 64 | -181  [-847 ; 484] | 165  [-500 ; 831] | 64  [-601 ; 730] | 110  [-554 ; 776] |  |
| ≥ 65 | 130  [-537 ; 797] | 477  [-190 ; 1144] | 376  [-290 ; 1044] | 423  [-244 ; 1089] | 312  [-352 ; 975] |

**Multimedia Appendix *3 (continued)***

| **Rush Back, ANOVA (F(5, 8994) = 239·8, p < 0·001, ω^2^ = 0·12)** | | | | | |
| --- | --- | --- | --- | --- | --- |
| Age group | 18 – 24 | 25 – 34 | 35 – 44 | 45 – 54 | 55 – 64 |
| 25 – 34 | **-1370**  **[-2075; -664]^$$$^** |  |  |  |  |
| 35 – 44 | **-3010**  **[-3715 ; -2304] ^$$$^** | **-1640**  **[-2345; -934] ^$$$^** |  |  |  |
| 45 – 54 | **-4730**  **[-5434; -4025] ^$$$^** | **-3360**  **[-4065; -2655] ^$$$^** | **-1720**  **[-2425; -1015] ^$$$^** |  |  |
| 55 – 64 | **-6267**  **[-6969; -5565] ^$$$^** | **-4897**  **[-5599; -4195] ^$$$^** | **-3257**  **[-3659; -2555] ^$$$^** | **-1537**  **[-2238; -836] ^$$$^** |  |
| ≥ 65 | **-6749**  **[-7453; -6044] ^$$$^** | **-5379**  **[-6083; -4674] ^$$$^** | **-3739**  **[-4443; -3054] ^$$$^** | **-2018**  **[-2722; -1315] ^$$$^** | **-481**  **[-1182; 219] ^$$$^** |

^$^ p < 0·05, ^$$^ p < 0·01 and ^$$$^ p < 0·001 after post-hoc Bonferroni’s corrections
