# Supplementary material for: Age-Associated Capacity to Progress When Playing Cognitive Mobile Games: Ecological Retrospective Observational Study
Source: JMIR Serious Games. 2020 Jun 12;8(2):e17121. doi: 10.2196/17121 (PMC7320308; doi:10.2196/17121)
Supplement: Multimedia Appendix 4 [file games_v8i2e17121_app4.docx]

## Multimedia Appendix 4: Results of the mixed effect models analysis. ß coefficient [95% CI]

| **Parameters** | | **Cognitive Mobile Game** | | | | | | |
| --- | --- | --- | --- | --- | --- | --- | --- | --- |
|  | | **Square Numbers** | **Memory Sweep** | **Word Pair** | **Babble Bots** | **Must Sort** | **Unique** | **Rush Back** |
| **p-value of the model** | | χ² (11) = 67 018,  P < 0·001 | χ² (11) = 30 123,  P < 0·001 | χ² (11) = 201 596,  P < 0·001 | χ² (11) = 54 202,  P < 0·001 | χ² (11) = 339 755,  P < 0·001 | χ² (11) = 315 917,  P < 0·001 | χ² (11) = 260 998,  P < 0·001 |
| **Fixed effect** | **Session** | 358  [351 ; 366] | 248  [244 ; 252] | 264  [259 ; 268] | 181  [178 ; 184] | 919  [911 ; 926] | 505  [500 ; 509] | 593  [588 ; 598] |
|  | **Age group** |  |  |  |  |  |  |  |
|  | 25 – 34 | 3155  [1937 ; 4373] | -968  [-1542 ; -395] | 942  [302 ; 1582] | 1755  [1049 ; 2462] | -559  [-1672 ; 552] | -400  [-1189 ; 388] | -1457  [-2133 ; -781] |
|  | 35 – 44 | 3005  [1788 ; 4221] | -5109  [-5682 ; -4537] | 1437  [798 ; 2076] | 966  [260 ; 1672] | -1710  [-2821 ; -599] | -3021  [-3809 ; -2233] | -2058  [-2733 ; -1383] |
|  | 45 – 54 | 2159  [944 ; 3374] | -8438  [-9009 ; -7867] | 1545  [907 ; 2182] | 181  [-524 ; 886] | -1958  [-3067 ; -849] | -7738  [-8525 ; -6951] | -3329  [-4003 ; 2655] |
|  | 55 – 64 | -799  [-2010 ; 411] | -11 990  [-12 559 ; -11 421] | 1288  [652 ; 1925] | -1119  [-1822 ; -416] | -2694  [-3800 ; -1589] | -10 315  [-11 098 ; -9531] | -4946  [-5618 ; -4275] |
|  | ≥ 65 | -4144  [-5385 ; -2930] | -14 214  [-14 782 ; -13 645] | 788  [151 ; 1425] | -3271  [-3975 ; -2567] | -3033  [-4138 ; -1928] | -12 879  [-13 664 ; -12 094] | -6797  [-7470 ; -6125] |

**Multimedia Appendix *4 (continued)***

|  | **Age^$^Session** |  |  |  |  |  |  |  |
| --- | --- | --- | --- | --- | --- | --- | --- | --- |
|  | 25 – 34 | -84  [-93 ; -76] | -99  [-104 ; -94] | -6  [-12 ; 1] | -47  [-50 ; -43] | -68  [-77 ; -59] | -79  [-84 ; -74] | -82  [-88 ; -76] |
|  | 35 – 44 | -147  [-155 ; -139] | -158  [-163 ; -153] | -44  [-49 ; -39] | -77  [-81 ; -73] | -305  [-314 ; -297] | -191  [-195 ; -186] | -280  [-285 ; -275] |
|  | 45 – 54 | -191  [-199 ; -183] | -207  [-212 ; -202] | -84  [-89 ; -79] | -102  [-106 ; -99] | -524  [-532 ; -516] | -270  [-275 ; -265] | -407  [-412 ; -401] |
|  | 55 – 64 | -231  [-239 ; -223] | -241  [-245 ; -237] | -111  [-116 ; 107] | -117  [-121 ; -113] | -683  [-690 ; -675] | -327  [-332 ; -322] | -478  [-482 ; -472] |
|  | ≥ 65 | -274  [-281 ; -266] | -250  [-254 ; -245] | -147  [-152 ; 143] | -124  [-128 ; -120] | -812  [-819 ; -804] | -392  [-397 ; -388] | -510  [-515 ; -505] |
|  | **Intercept** | 10 576  [9 711 ; 11 442] | 14 579  [14 169 ; 14 988] | 4642  [4186 ; 5098] | 4248  [3747 ; 4749] | 4283  [3494 ; 5073] | 19 025  [18 466 ; 19584] | 8724  [8245 ; 9203] |
| **Random effect** | **μ** | 16 656  [16 412 ; 16 904] | 7619  [7503 ; 7736] | 8663  [8535 ; 8792] | 9607  [9466 ; 9750] | 15 028  [14 807 ; 15 523] | 10 801  [10 643 ; 10 962] | 9192  [9056 ; 9329] |
|  | **σ** | 12 180  [12 157 ; 12 202] | 7818  [7801 ; 7836] | 7658  [7644 ; 7672] | 10 242  [10 226 ; 10 258] | 16 283  [16 255 ; 16 312] | 8527  [8511 ; 8542] | 8201  [8185 ; 8216] |
